# Examining Predictors of Early Admission and Transfer to the Critical Care Resuscitation Unit

**DOI:** 10.5811/westjem.58356

**Published:** 2023-06-28

**Authors:** Quincy K. Tran, Daniel Najafali, Tiffany Cao, Megan Najafali, Nelson Chen, Iana Sahadzic, Ikram Afridi, Ann Matta, William Teeter, Daniel J. Haase

**Affiliations:** * University of Maryland School of Medicine, Department of Emergency Medicine, Baltimore, Maryland; † University of Maryland School of Medicine, The R Adams Cowley Shock Trauma Center, Baltimore, Maryland; ‡ University of Maryland School of Medicine, The Research Associate Program in Emergency Medicine and Critical Care, Department of Emergency Medicine, Baltimore, Maryland; § University of Maryland School of Medicine, Baltimore, Maryland; || University of Illinois Urbana-Champaign, Carle Illinois College of Medicine, Champaign, Illinois; # University of Maryland Medical Center, The Critical Care Resuscitation Unit, Baltimore, Maryland

## Abstract

**Introduction:**

Previous studies have demonstrated that rapid transfer to definitive care improves the outcomes for many time-sensitive conditions. The critical care resuscitation unit (CCRU) improves the operations of the University of Maryland Medical Center (UMMC) by expediting the transfers and resuscitations for critically ill patients who exceed the resources at other facilities. In this study we investigated CCRU transfer patterns to determine patient characteristics and logistical factors that influence bed assignments and transfer to the CCRU. We hypothesized that CCRU physicians prioritize transfer for critically ill patients. Therefore, those patients would be transferred faster.

**Methods:**

We performed a retrospective review of all non-traumatic adult patients transferred to the CCRU from other hospitals between January 1–December 31, 2018. The primary outcome was the interval from transfer request to CCRU bed assignment. The secondary outcome was the interval from transfer request to CCRU arrival. We used multivariate logistic regressions to determine associations with the outcomes of interest.

**Results:**

A total of 1,741 patients were admitted to the CCRU during the 2018 calendar year. Of those patients, 1,422 were transferred from other facilities and were included in the final analysis. Patients’ mean age was 57 ± 17 years with a median Sequential Organ Failure Assessment (SOFA) score of 3 [interquartile range 1–6]. Median time from transfer request to CCRU bed assignment was 8 (0–70) minutes. A total of 776 (55%) patients underwent surgical intervention after arrival. Using the median transfer request to bed assignment time, we found that patients requiring stroke neurology (odds ratio [OR] 5.49, 95% confidence interval [CI] 2.85–10.86), having higher SOFA score (OR 1.04, 95% CI 1.001–1.07), and needing an immediate operation (OR 2.85, 95% CI 1.98–4.13) were associated with immediate bed assignment time (≤8 minutes). Patients who were operated on (OR 0.74, 95% CI 0.55–0.99) were significantly less likely to have an immediate bed assignment time.

**Conclusion:**

The CCRU expedited the transfer of critically ill patients who needed urgent interventions from outside facilities. Higher SOFA scores and the need for urgent neurological or surgical intervention were associated with near-immediate CCRU bed assignment. Other institutions with similar models to the CCRU should perform studies to confirm our observations.

## INTRODUCTION

When caring for a patient exceeds the initial hospital’s resources, patients are usually transferred to tertiary or quaternary medical centers for higher levels of care. The transfer of patients from either the emergency department (ED) or intensive care unit (ICU) to regional referral centers, interhospital transfer (IHT), is becoming more common.^1^,^2^ However, the system for medical IHT is not robust and most of the high-volume referral medical centers do not have immediate ICU bed availability.^3^,^4^ As a result, the IHT of critically ill patients is often delayed, leading to worse patient outcomes.^5^

Furthermore, certain patients are found to have time-sensitive disease and will need transfer to tertiary or quaternary care centers for immediate diagnostic or therapeutic interventions. Early intervention or early arrival at referral centers have been associated with improved outcomes for patients who had Type A aortic dissection, ruptured abdominal aortic aneurysm, intracranial hypertension, or ischemic stroke from large vessel occlusion.^6^–^9^

To expedite IHT and optimize outcomes for patients with critical illness or time-sensitive conditions, the critical care resuscitation unit (CCRU) at the University of Maryland Medical Center (UMMC) was created in July 2013. The CCRU streamlines the IHT process by providing timely and effective resuscitation for patients who need further interventions from a quaternary care medical center. The CCRU has been found to outpace traditional ICUs by providing care for a higher number of patients with critical illnesses or time-sensitive diseases, while also leading to improved outcomes.^4^,^10^ However, details about how the CCRU physicians triage and prioritize transfer requests while optimizing patient volume has not been described. In this study we aimed to investigate and describe factors that contribute to early bed assignment and early transfer to the CCRU. Determining these factors can provide further information for administrators at other facilities that might be interested in setting up a similar unit.

## METHODS

### Study Design and Clinical Setting

We performed a retrospective analysis of data from all adult patients who were transferred from other hospitals to the CCRU between January 1–December 31, 2018 (the latest year that this data became available). All non-traumatic adult patients who were admitted from other hospitals to the CCRU during this period were eligible. We excluded patients who were admitted to the CCRU from within our medical center (intrahospital transfer), either from our institution’s ED or another inpatient unit. This study was exempt from formal consent by our institutional review board (HP-00084554).

The CCRU is a six-bed, ICU-based resuscitation unit that is staffed around the clock by one attending physician, one advanced practice provider (APP), and three to four nurses who are required to have at least two years of prior ICU experience. The operational costs to staff the CCRU, represented in full-time equivalents (FTE), are 5.0 FTE for APPs, 5.0 FTE for attending physicians, and 32.0 FTE for nurses. When a referring clinician needs to transfer a patient with critical illness or time-sensitive disease to our quaternary medical center, the referring clinician first contacts the Maryland ExpressCare (MEC) Center, which handles all transfer logistics for our institution. The MEC personnel then contact the on-call specialty physician and the appropriate ICU physician for possible transfer. When no ICU bed is available, the CCRU attending physician is involved with the transfer request for an available CCRU bed.

**Population Health Research Capsule**

What do we already know about this issue?

*Interhospital transfers of critically ill patients are often delayed as there is no immediate ICU bed availability*.

What was the research question?

*What are the transfer patterns and patient characteristics that lead to immediate bed assignment at the critical care resuscitation unit (CCRU)?*

What was the major finding of the study?

*Patients admitted to stroke neurology, with higher SOFA score, and needing immediate surgery, were associated with immediate bed assignment time (*≤*8 minutes)*.

How does this improve population health?

*A CCRU expedites the transfer of critically ill patients from outside facilities who are in need of urgent intervention*.

Once the patient is considered appropriate for transfer by the specialist and the CCRU attending physician, the CCRU attending assigns an available bed to the patient, as appropriate. This assigned bed is now reserved until this patient arrives. Once a bed is assigned to the patient, the CCRU team and the accepting physicians at UMMC leave the decision of transport to the referring clinicians. The sending facility will arrange for the most appropriate mode of transport (eg, ground vs air transport, private vs academic-affiliated transport teams) for the patient, according to the transport team’s availability and the patient’s acuity.

The CCRU is a fluid and dynamic unit. Regarding the capacity of the CCRU, “empty” beds may already be promised to a patient in transit; thus, even though a patient is not physically occupying the bed, it is technically not empty. The CCRU attendings triage patients’ acuity levels and the demands of the unit for appropriate bed assignment. There is an agreement within the medical center that when the CCRU is reaching its capacity that any available bed in the medical center will be given to a CCRU patient. Typically, when the CCRU is reaching its capacity, such as a situation where five of the six beds are occupied, the CCRU calls the appropriate inpatient unit(s) to alert them, so that the CCRU can get their first available bed when it becomes available.

Once a patient arrives at the CCRU, the patient will receive resuscitation from the CCRU team as indicated and timely diagnostics or therapeutic intervention(s) from specialists, such as a thrombectomy for ischemic stroke from large vessel occlusion, or cannulation for extracorporeal membrane oxygenation (ECMO), etc. In addition, patients may be taken to the operating room (OR) for emergent surgery, which was defined as within 12 hours of CCRU arrival.^4^ Once a patient undergoes adequate resuscitation and treatment by the multidisciplinary clinical teams, the patient will be moved to another appropriate inpatient unit at our medical center for further longitudinal care.

### Data Collection

We extracted the following data from the CCRU records and our institution’s electronic health records: the date and time that the referring clinicians contacted MEC for a transfer request; the date and time that the CCRU attending physicians assigned a bed to each patient; and the date and time that patients arrived at the CCRU. We also extracted other clinical data, such as components of the Sequential Organ Failure Assessment (SOFA) score upon a patient’s arrival at the CCRU. The SOFA score served as a surrogate for a patient’s disease severity at the sending facility, prior to arrival at the CCRU. We also collected other laboratory values that were not part of the SOFA score, such as serum lactate concentrations, white blood cell count, and hemoglobin concentrations. We collected the types of continuous infusions prior to CCRU arrival, such as insulin and anti-hypertensive infusions, as a marker of care intensity at the referring facilities.

The UMMC is a quaternary medical center with a capacity of approximately 800 beds. There are five adult non-trauma ICUs: the cardiac surgical ICU; coronary care unit; neurocritical care unit; medical ICU (MICU); and surgical ICU. UMMC has a system in place to handle transfer requests from another hospital via MEC. MEC works to connect the transferring clinicians with the relevant ICU for possible admission. If patients are deemed appropriate for admission but the corresponding ICU does not have available beds, the CCRU will be included in the consultation. The transfer records of patients coming from another hospital to UMMC are maintained by MEC as part of their operations, which manages all incoming IHT.

We extracted the data used from non-CCRU units in this study from the MEC database. Prior to commencement of data extraction, investigators who were blinded to the study’s hypothesis were trained by the principal investigator with sets of 10 patients until data accuracy reached at least 90% agreement. Data was extracted into a standardized Excel spreadsheet (Microsoft Corporation, Redmond, WA). Up to 5% of data was double-checked by an independent investigator to maintain inter-rater agreement of ≥90%. The data abstracters were blinded to the study hypothesis and objectives. Our study adhered to the reporting practices outlined by Worster et al.^11^ Our study also met the minimum requirements outlined by Lowenstein’s editorial regarding medical records review in emergency medicine.^12^ If a component of the SOFA score was missing, we imputed a normal value.

### Outcome Measures

The primary outcome was the percentage of patients who were immediately assigned a bed at the CCRU (≤8 minutes) from time of transfer request. Our secondary outcome was the percentage of patients who arrived early to the CCRU, which was defined by the median time interval from transfer request to CCRU arrival (<180 minutes). An additional outcome investigated was mortality of patients transferred to the CCRU.

### Statistical Analysis

We did not perform a sample-size calculation for this descriptive study, presuming that we would obtain an adequate sample size for our analysis by using a full calendar year of CCRU admissions. Based on a previous study, it was estimated that we would have approximately 1,400 patients using a full calendar year of CCRU admissions.^10^ By using the median time from transfer request to bed assignment or CCRU arrival as cut-off points to dichotomize our primary and secondary outcomes, we estimated that we would have approximately 700 patients for each of our outcomes. This would provide enough of a sample size to accommodate multivariable logistic regressions with at least 70 independent variables, according to the previous recommendation of 10 counts of outcome events per each independent variable.^13^

We used descriptive analyses to present patients’ demographic and clinical information with mean (±SD or median (interquartile range [IQR]), or frequency (percentage) as appropriate. Continuous variables between groups were compared with the Student *t*-test or the Mann-Whitney U test, while categorical variables were compared with the Pearson chi-squared test or Fisher exact test, as indicated. We performed multivariate logistic regressions with dichotomous outcomes as immediate CCRU bed assignment (vs normal CCRU bed assignment), early CCRU arrival (vs normal CCRU arrival), or deceased (vs living). All relevant independent variables were determined a priori and were used in the regression models.

Prior to data analysis, we performed preliminary analyses and defined immediate CCRU bed assignment as the median of the time interval from transfer request to CCRU bed assignment as the cut-off time for our dichotomous outcome (immediate vs normal CCRU bed assignment). Similarly, the median time interval from transfer request to CCRU arrival was the cut-off point for the dichotomous outcome of early CCRU arrival vs normal CCRU arrival. We assessed multicollinearity of the models using variance inflation factor (VIF). A threshold of VIF ≥5 was used to deem any factor as having multicollinearity. Any independent variable with VIF ≥5 would meet the threshold for having high collinearity and would be excluded from the multivariable logistic regression. No independent variable met this threshold in any of our models. All models were assessed for goodness of fit using the Hosmer–Lemeshow test. A *P* > 0.05 was the threshold indicating that the model was an appropriate fit.

We performed all statistical analyses with Minitab version 19.0 (Minitab, LLC, State College, PA) or using R version 4.1.0 (R Project for Statistical Computing, Vienna, Austria) and RStudio version 1.4.1717 software (RStudio PBC, Boston, MA). All two-sided *P*-values < 0.05 were considered statistically significant.

## RESULTS

### Patient Demographics of All UMMC Facilities and Patients Triaged to the CCRU

**Figure 1:**
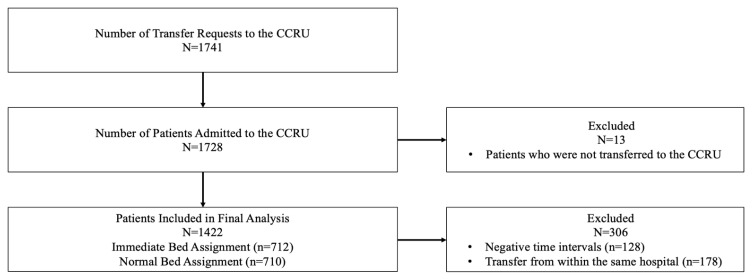
Patient selection diagram outlining patients included in the final analysis. *CCRU*, critical care resuscitation unit.

We identified 1,741 patients who were admitted to the CCRU during the study period, and 1,422 patients who were transferred from other hospitals with sufficient data included in our final analysis (Figure 1). There were 712 (50%) patients who had immediate bed assignment to the CCRU, defined as within eight minutes. There were 750 (53%) patients who arrived at the CCRU within 180 minutes from the time of transfer request, while the majority of patients (1,168, 82%) arrived at the CCRU within 360 minutes from the time of transfer request.

Demographic data are reported in Table 1A for the 5,717 patients from all UMMC units comprised of the following: CCRU; any adult, non-trauma ICU; ED; and other inpatient units (intermediate care unit or surgical or medical wards). Patients who were transferred to the CCRU had the shortest time interval from transfer request to bed assignment, with a median (IQR) of 8 [0–70] minutes (*P* < 0.001), when compared to patients who were transferred to other inpatient units at our medical center. When stratifying patients by admission location (CCRU vs ICU vs ED vs other inpatient units), the CCRU had statistically significant higher rates of air transport compared to other units (17% CCRU vs 10% ICU vs 1% ED, vs 4% other inpatient units, *P* < 0.001).

The CCRU patients’ demographic data are reported in Table 1B stratified by bed assignment time (immediate vs normal). Patients who were immediately assigned a CCRU bed also arrived at the CCRU faster than those who received a normal bed assignment time (Table 1C). Patients with normal bed assignment time had a statistically lower rate of initiation of mechanical ventilation (37% immediate vs 29% normal, *P* = 0.002) and lower proportion receiving anti-hypertensive infusion, when compared with patients who had immediate bed assignment time (15% immediate vs 10% normal, *P* = 0.005). Among all patients who underwent surgical interventions, a greater number of patients with normal bed assignment time underwent surgery during hospitalization at UMMC (58% normal vs 51% immediate, *P* = 0.009), but a significantly higher proportion of patients with immediate bed assignment were taken to the OR within 12 hours compared to those patients who had normal bed assignment (27% immediate vs 20% normal, *P* = 0.002). More patients with normal bed assignment were discharged home and survived compared to patients with immediate bed assignment time (44% normal vs 38% immediate, *P* = 0.014).

### Primary Outcome: Transfer Request to Bed Assignment

**Figure 2:**
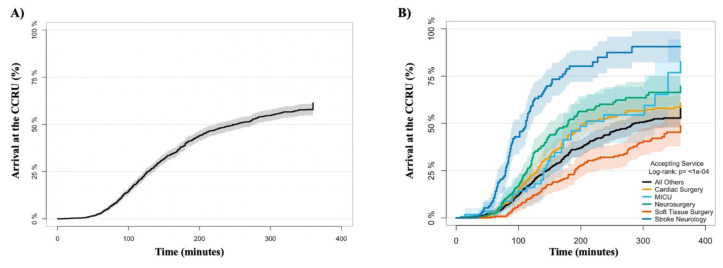
Kaplan-Meier curve for bed assignment to the CCRU (A) Time intervals for bed assignment to the CCRU for all patients. (B) Comparison of time intervals for bed assignment to the CCRU based on accepting service. * The 50% mark indicates the censored time. *CCRU*, critical care resuscitation unit; *MICU*, medical intensive care unit.

**Figure 3:**
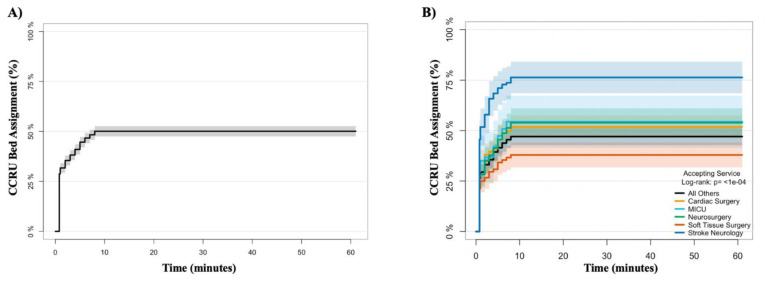
Kaplan-Meier curve for arrival at the CCRU: (A) Time intervals for all patients arriving to the CCRU. (B) Comparison of time intervals from arrival to the CCRU based on accepting service. *CCRU*, critical care resuscitation unit; *MICU*, medical intensive care unit.

The Kaplan–Meier curves presented in Figure 2A and Figure 2B depict each time interval from transfer request to CCRU bed assignment. The top five accepting services based on volume for the CCRU during the 2018 calendar year were as follows: 1) cardiac surgery (297, 21%); 2) soft tissue surgery (240, 16%); 3) neurosurgery (191, 13%); 4) acute care emergency services (147, 10%); and 5) vascular surgery (134, 9%). Appendix 1 provides a complete list of accepting services for patients being transferred to the CCRU. Figure 2B illustrates the CCRU bed assignment at each time interval from transfer requests for respective accepting services. Figure 3A demonstrates the time from transfer request and that ~50% of patients arrive at the CCRU within three hours. The time interval that patients arrive at the CCRU stratified by accepting service is represented in Figure 3B.

The covariates that were significantly associated with our primary outcome of interest—transfer request to bed assignment ≤8 minutes—are reported in Table 2. The full multivariate model is reported in Appendix 2. Patients requiring stroke neurology (odds ratio [OR] 5.49, 95% confidence interval [CI] 2.85–10.86, *P* < 0.001), with an increased SOFA score (OR 1.04, 95% CI 1.00–1.07, *P* = 0.047), and receiving surgical operation within 12 hours (OR 2.85, 95% CI 1.98–4.13, *P* < 0.001) were associated with immediate bed assignment time. However, patients who would receive any surgical intervention during hospitalization (OR 0.74, 95% CI 0.55–0.99, *P* = 0.044) were significantly less likely to have an immediate bed assignment time.

### Secondary Outcome: Transfer Request to Arrival at the Critical Care Resuscitation Unit

We reported the statistically significant findings from our multivariate analysis of our secondary outcomes in Table 2 and the full multivariate models are available in Appendix 3. Factors significantly associated with increased likelihood of transfer request to CCRU arrival time <180 minutes were patients being accepted by stroke neurology (OR 12.81, 95% CI 6.09–28.51, *P* < 0.001) and being taken to the OR for emergent surgical intervention (OR 3.15, 95% CI 2.12–4.71, *P* < 0.001). Higher SOFA score (OR 0.96, 95% CI 0.92–0.99, *P* = 0.033) was associated with decreased likelihood of arrival within 180 minutes.

### Other Outcome: Predictors for Mortality Among Patients Transferred to the CCRU

The statistically significant covariates are presented in Table 2 for the outcome of mortality and the result of the complete multivariate logistic regression is available in Appendix 3. Accepting services associated with a significant likelihood of mortality were neurosurgery (OR 4.14, 95% CI 1.87–9.37, *P* < 0.001) and stroke neurology (OR 3.00, 95% CI 1.20–7.52, *P* = 0.019). Each gram per deciliter increase in hemoglobin was protective and associated with 12% decreased risk of death (OR 0.88, 95% CI 0.80–0.95, *P* = 0.002).

All of the model’s Hosmer–Lemeshow goodness of fit tests returned *P* > 0.05, indicating the data fit the models well, and all VIF values for each covariate were <5 indicating that multicollinearity was not present.

## DISCUSSION

When originally conceptualized, the purpose of the CCRU—similar to the model of trauma transfer—was to provide “immediate ICU access to accommodate IHT with time-sensitive surgical critical illness,” while “decreasing lost admissions, minimizing transfer times, and improving outcomes of known trauma critical care transfers.”^4^ Having demonstrated a significant increase in the volume of transfers, decreased transfer times, and decreased time to operative intervention, the CCRU expanded its patient population, including other critical care emergencies requiring intervention, such as acute ischemic stroke caused by large vessel occlusion and coronary interventions.^4^,^9^,^10^ While formalized systems exist for patients transferred between hospitals with traumatic etiologies, ST-segment elevation myocardial infarction and stroke, there are no formal systems to ensure timely transfer for other highly morbid, highly fatal, and time-sensitive conditions, including acute aortic diseases, toxicologic, obstetric, septic, respiratory, and other vascular emergencies. The CCRU demonstrates that such a regionalized ICU is feasible, although further studies are necessary to elucidate how units like the CCRU could affect the operations of referring hospitals.

In general, the literature supports the conclusion that patients with critical illness or time-sensitive diseases should be referred to large-volume centers with multiple advanced modalities and therapeutic options that are not available at most acute care hospitals.^14^ However, bed availability is usually a prime factor in the delay of care for these patients as ICU bed utilization in most hospitals is not oriented toward the acceptance of outside transfers. Furthermore, there is no formal system to triage and prioritize the transfer of these patients. Therefore, immediate acceptance to an ICU bed is difficult for most units, and a significant amount of time and uncertainty is lost in the interim between initial consult and eventual bed assignment. A traditional ICU may need to decide whether to leave a bed open, while other critically ill patients wait in the ED or other part of the hospital. On the other hand, a traditional ICU may fill all the available beds and then waitlist patients from other hospitals.

The CCRU offers solutions to avoid many problems that traditional ICUs face when they are confronted with an emergent bed request. One of our guiding principles is to maintain bed availability continuously for emergent transfer requests for patients with all disease states. The CCRU has been largely successful in being able to accommodate appropriate requests for emergent transfer by maintaining effective flow management through the entire system with institutional support.

In this study we investigated the triage process and the prioritization of patients who needed to be transferred for higher levels of care. We evaluated patient characteristics and logistical factors associated with rapid acceptance and transfer to the CCRU by comparing those patients with immediate assignment vs those who waited a more standard time for bed assignment after initial consult. Not surprisingly, those patients with immediate bed assignment were more likely to have a more rapid arrival, arrive by air, and undergo surgical interventions within 12 hours of arrival. These patients also had higher SOFA scores and were more likely to be mechanically ventilated or receiving continuous infusions. All these characteristics are arguably markers found in those illnesses requiring more time-sensitive intervention, resuscitation, or higher care intensity and, therefore, highest priority for immediate bed assignment. Furthermore, the time-sensitive nature for those patients who require timely intervention and resuscitation is likely the main driver of early transfer and would seemingly affect their outcomes.

The triage and prioritization process for early CCRU bed assignment and transfer may have explained previous observations regarding critically ill patients’ outcomes. Previously, we have shown that patients requiring higher critical care intensity who were transferred from other hospitals’ EDs to the CCRU were associated with lower likelihood of mortality when compared with those admitted to other traditional adult non-trauma ICUs in our own medical center. 10 Similarly, patients who received early intervention at an ED-based resuscitation unit were also associated with lower 30-day mortality. 15 Thus, our triage process eliminated lost time and uncertainty for the referring clinicians, while potentially optimizing patients’ outcomes.

In this study, the severity and characteristics of patients requiring immediate bed assignment were largely supported by the primary and secondary outcome analyses. Criteria such as the severity of illness, as indicated by high SOFA scores, needing operative intervention within 12 hours, and admission of these patients to the stroke neurology service at a comprehensive stroke center, are plausible factors to necessitate immediate bed assignment. Similarly, when predicting which patients would be associated with a higher burden of morbidity and mortality, those with emergent need for neurosurgical and stroke intervention seem natural candidates and an intuitive choice. Patients with strokes have a time-sensitive disease if the patient is a candidate for mechanical thrombectomy (eg, occlusion in a large vessel such as the middle cerebral artery or internal carotid artery); thus, they would be prioritized by the CCRU team during triage.

Minimizing the time from symptom onset to reperfusion for candidates of mechanical thrombectomy is known to impact patient outcomes; thus, the CCRU is designed to expedite transfer for these patients in conjunction with our comprehensive stroke center.^16^ Hemorrhagic stroke would also be another reason patients are prioritized if they require interventions to prevent increased intracranial pressure. Furthermore, severity of illness is directly linked to mortality; therefore, it is not surprising that arrival SOFA score, high serum lactate, age, and troponin would be the multivariate factors predictive for mortality, as each can reasonably be associated with mortality itself in undifferentiated critical illness.

The time intervals from transfer request to CCRU arrival for the early arrival group is multifactorial. As our institution is a comprehensive stroke center, it is not surprising that the strongest factor for a patient arriving rapidly to our medical center is for emergent intervention by the stroke neurology team. Similarly, those patients needing emergent evaluations and interventions by neurosurgery, vascular surgery, and cardiac surgery, as well as other patients requiring any medication infusion and those resulting in operative intervention within 12 hours would be priorities for transfer in most situations. On the other hand, those patients who were requested for transfer during daylight hours would theoretically undergo early surgical interventions upon arrival, and these patients would also benefit from the higher nursing staffing and resources available when compared to nighttime levels at most institutions.^17^

Given the complexity and myriad of factors that play a role in the transport of patients to our center, distance and ground transport negatively affecting the intervals between transfer request and arrival time are appropriate. Similarly, patients who would need to be transferred for non-urgent but complex surgical interventions that are only available at a quaternary medical center may not need to be transferred immediately, thereby explaining how those patients undergoing any operative intervention during their hospitalization at our medical center did not arrive at the CCRU within 180 minutes. However, patients having higher arrival SOFA score, but not an elevated serum lactate, did not arrive to the CCRU early. This was a curious and counterintuitive finding that warrants further study. It is probable that patients who were critically ill with high SOFA scores were associated with prolonged stabilization before patients could be stable for transfer; thus, it could have taken them longer to be transferred, although a bed was already ready for them.^18^,^19^

Single elevation of lactate could be multifactorial from several conditions, including but not limited to status epilepticus or hypovolemia. Therefore, those patients can be evaluated along the way with resuscitation at the referring center. Typically, lactate can be cleared with prompt and effective treatment. Upon evaluation of a patient for transfer to the CCRU, a CCRU attending asks about factors such as ventilator mode, arterial blood gas(es), resulted laboratory values, and vasopressor requirements, among other factors, so that a mental picture can be created of the patient’s SOFA score prior to arrival. The SOFA score is a composite metric that involves a patient’s neurologic, cardiac, renal, hematologic, and hepatic status. All components of the SOFA score will be asked at the time of transfer request and then an idea will be formed of how critically ill the patient is. This will then become more objective when a SOFA score is calculated when the patient arrives. Based on the clinical picture of the patient, the CCRU attendings will prioritize how soon a patient will come to the CCRU and their bed assignment time. Lactate does play a role in prioritization but only if lactate is elevated and caused by a condition that requires intervention at a quaternary care center such as elevation caused by an ischemic limb, ischemic bowel, or severe acute respiratory distress syndrome requiring ECMO, among other conditions.

One peculiarity throughout the modeling is the negative association with any operative intervention or acceptance by our soft tissue surgery service. This potential inconsistency is likely a result of our policy for the admission and management of patients with soft tissue infection. The R Adams Cowley Shock Trauma Center at the University of Maryland is a regional referral center for soft tissue infections and surgery. During the day between 7 am and 5 pm, all surgical operations and transfer requests are handled by the regular soft tissue surgery team, which is staffed by our trauma surgeons who specialize in soft tissue infection. The service is covered during overnight hours by the on-call trauma service.

To avoid taking the attention of trauma surgeons away from the critically ill trauma patients, our clinical practice is to admit all patients with soft tissue infections to the CCRU first, thus allowing our critical care staff to evaluate and manage these complex patients. Unless these patients need urgent surgical intervention, which the trauma surgeons will carry out, these patients will otherwise undergo scheduled operations, as clinically indicated, at a later day with the regular soft tissue surgeons. Therefore, while the acuity of those patients who have necrotizing fasciitis would be high and would require urgent interventions and immediate bed assignment, most patients with soft tissue infections do not require immediate surgical intervention at arrival, either because they have received an initial debridement at the referring hospital or they have an intermediate acuity disease state (eg, cellulitis, abscess, etc) that warrants longer wait times for transport and subsequent surgical interventions at a later time. Nevertheless, the patients with soft tissue infections, which represent approximately 13% of our population, could partly explain these observations. Future studies specifically involving patients with soft tissue infection are needed to further investigate this phenomenon.

## LIMITATIONS

Our study has several strengths, as well as some limitations that should be highlighted. We only focused on the patients who were accepted to the CCRU; we did not have data regarding patients who were not accepted to the CCRU. When the CCRU reaches its capacity and immediate bed assignment is not possible, referring clinicians may opt to contact other tertiary facilities to transfer their patients. Our study involved a large and heterogenous patient population; therefore, we had to categorize patients according to the accepting services and could not identify the individual disease states that would necessitate immediate bed assignment. For example, all patients who were admitted to our medical intensive care unit were categorized as being accepted by the pulmonary and critical care service. These patients could span a variety of conditions, such as respiratory failure, gastrointestinal bleeding, septic shock, etc. Finally, we used the SOFA score as a surrogate marker for disease severity, but it does not apply to all disease states, such as patients with stroke or spontaneous intracerebral hemorrhage.

## CONCLUSION

Our study showed that patients who had high acuity or required urgent surgical intervention, such as patients with ischemic stroke, were given highest priority to have immediate bed assignments at the CCRU. However, patients who had high SOFA scores or were referred during overnight hours were associated with longer intervals between transfer request to arrival at the CCRU. Further studies are necessary to confirm our observations and to investigate the relationship between this group of patients and their outcomes.

## Supplementary Information



## Figures and Tables

**Table 1A:** Demographics and clinical features of patients from all University of Maryland Medical Center units in 2018.

Variables	All UMMC*	UMMC CCRU	UMMC ICU**	UMMC ED	UMMC other inpatient units	P†
Total patients, N	5,717	1,422	1,046	817	2,432	NA
Age (years), mean (SD)	56 (18)	57 (17)	59 (16)	48 (18)	54 (17)	**< 0.001, < 0.001, < 0.001**
Type of transport, N (%)^						
Air	443 (8)	240 (17)	103 (10)	10 (1)	90 (4)	**< 0.001, < 0.001, < 0.001**
Ground	5134 (90)	1182 (83)	941 (90)	716 (88)	2295 (94)	**< 0.001, < 0.001, < 0.001**
Unknown	140 (2)	0 (0)	2 (0)	91 (11)	47 (2)	
Ground distance (km), median [IQR]	44 [11–75]	44 [13–75]	44 [13–74]	21 [8–64]	44 [13–92]	**0.035, < 0.001**, 0.68
Type of referring hospital, N (%)^						
Teaching	1,837 (32)	460 (32)	379 (36)	233 (28)	765 (31)	**0.037, 0.041**, 0.72
Community	3,663 (64)	962 (68)	663 (63.5)	397 (49)	1641 (68)	
Other/unknown	217 (4)	0 (0)	4 (0.5)	187 (23)	26 (1)	**0.005, < 0.001, < 0.001**
Transfer request to placement (min), median [IQR]	88 [15–410]	8 [0–70]	243 [77–660]	20 [10–53]	221 [50–1055]	**< 0.001, < 0.001, < 0.001**
Admission day of the week, N (%)						
Weekday (Monday–Friday)	4,323 (76)	1,046 (74)	806 (77)	538 (66)	1933 (79)	**0.047, < 0.001, < 0.001**
Weekend (Saturday–Sunday)	1394 (24)	376 (26)	240 (23)	279 (34)	499 (21)	
Admission time of the day, N (%)						
Day time (7:00 AM–7:00 PM)	3,881 (68)	679 (48)	801 (77)	496 (61)	1905 (78)	**< 0.001, < 0.001, < 0.001**
Evening time (7:01 PM–06:59 AM)	1,836 (32)	743 (52)	245 (23)	321 (39)	527 (22)	
Accepting service, N (%)^						
Emergency general surgery	223 (4)	147 (10)	1 (0)	0 (0)	75 (3)	
Cardiac surgery	732 (13)	297 (21)	106 (10)	0 (0)	329 (14)	
Cardiology	906 (16)	12 (1)	373 (36)	0 (0)	521 (21)	
Neurology	317 (6)	114 (8)	145 (14)	0 (0)	58 (2)	
Neurosurgery	392 (7)	191 (13)	164 (16)	0 (0)	37 (2)	**< 0.001**, NA, **< 0.001**
Oncology	208 (4)	2 (1)	0 (0)	0 (0)	206 (8)	
Pulmonary and critical care	311 (5)	57 (4)	252 (14)	0 (0)	2 (0)	**< 0.001, < 0.001, < 0.001**
Thoracic surgery	99 (2)	25 (2)	0 (0)	0 (0)	74 (3)	
Transplant	334 (6)	38 (3)	3 (0)	0 (0)	293 (12)	
Vascular surgery	224 (4)	134 (9)	1 (0)	0 (0)	89 (4)	
Other accepting services	1,971 (34)	405 (28)	1 (0)	817 (100)	748 (31)	

* All UMMC does not include pediatric or trauma patients.

** ICU patients are separate from CCRU.

^ Indicates that the top group of P-values was calculated excluding the unknown or other/unknown group and that the bottom group of P-values was calculated with the unknown or other/unknown groups with either Pearson chi-square test or Fisher exact test as appropriate.

† Bold cells indicate statistically significant findings (P < 0.05).

P-values written as P_1_, P_2_, P_3_ where: P_1_ = CCRU vs. ICU; P_2_ = CCRU vs. ED; P_3_ = CCRU vs. Other Inpatient Units

*CCRU*, critical care resuscitation unit; *ED*, emergency department; *ICU*, intensive care unit; *IQR*, interquartile range; *min*, minutes; *km*, kilometers; *NA*, not applicable; *UMMC*, University of Maryland Medical Center.

**Table 1B:** Demographics of accepted patients triaged to the critical care resuscitation unit.

Variables	All patients	Immediate bed assignment (≤ 8Minutes)	Normal bed assignment (>8 Minutes)	P†
Total patients, N	1,422	712	710	NA
Age (years), mean (SD)	57 (17)	58 (17)	57 (16)	0.72
Gender, N (%)				
Male	783 (55)	378 (53)	405 (57)	0.13
Female	639 (46)	334 (47)	305 (43)	
Past medical history, N (%)				
HTN	644 (45)	326 (46)	318 (45)	0.71
DM	347 (24)	165 (23)	182 (26)	0.28
Any liver disease	99 (7)	45 (6)	54 (8)	0.34
Any kidney disease	238 (17)	114 (16)	124 (17)	0.46
Any heart disease	315 (22)	157 (22)	158 (22)	0.93
Type of referring hospital, N (%)				
Teaching hospital	460 (32)	237 (33)	223 (31)	0.45
Non-teaching hospital	962 (68)	475 (67)	487 (69)	
Ground distance (km), mean (SD)	55 (56)	56 (58)	54 (54)	0.45
Transport by air, N (%)	240 (17)	157 (22)	83 (12)	**< 0.001**
Transfer request details, N (%)				
Transfer request weekdays (Mon–Fri)	1046 (74)	517 (73)	529 (75)	0.42
Transfer request weekend (Sat–Sun)	376 (26)	195 (27)	181 (25)	
Transfer request at night	743 (52)	313 (44)	430 (61)	**< 0.001**
Transfer request weekend night	208 (15)	103 (14)	105 (15)	0.86
Laboratory values				
WBC count (counts/μL), mean (SD)	14.28 (15.51)	14.60 (19.70)	13.96 (9.78)	0.44
Hemoglobin (g/dL), mean (SD)	11.5 (4.3)	11.7 (3.5)	11.4 (5.0)	0.13
Serum lactate (mmol/dL), mean (SD)	2.30 (2.32)	2.49 (2.59)	2.11 (2.00)	**0.002**
First troponin level (ng/L), median [IQR]	0.020 [0.010–0.110]	0.020 [0.010–0.120]	0.020 [0.010–0.100]	0.47
SOFA score, median [IQR]	3 [1–6]	3 [1–7]	2 [1–6]	**0.001**

† Bold cells indicate statistically significant findings (P < 0.05).

*CCRU*, critical care resuscitation unit; *dL*, deciliter; *DM*, diabetes mellitus; *Fri*, Friday; *g*, gram; *HTN*, hypertension; *IQR*, interquartile range; *km*, kilometer; *μL*, microliter; *mmol*, millimole; *Mon*, Monday; *ng*, nanogram; *NA*, not applicable; *Sat*, Saturday; *SOFA*, Sequential Organ Failure Assessment; *SD*, standard deviation; *Sun*, Sunday; *WBC*, white blood cell.

**Table 1C:** Clinical characteristics of accepted patients triaged to the critical care resuscitation unit.

Variables	All patients	Immediate bed assignment (≤ 8Minutes)	Normal bed assignment (>8 Minutes)	P†
Total patients, N	1,422	712	710	NA
Transfer request to bed assignment (min), median [IQR]	8 [0–70]	0 [0–3]	70 [22–254]	**< 0.001**
Transfer request to arrival (min), median [IQR]	174 [115–290]	131 [93–183]	253 [163–447]	**< 0.001**
CCRU LOS (hours), median [IQR]	7 [4–18]	6 [3–15]	8 [4–19]	**< 0.001**
Continuous infusion, N (%)				
Any anti-hypertensive	184 (13)	110 (15)	74 (10)	**0.005**
Insulin	189 (13)	92 (13)	97 (14)	0.68
Invasive mechanical ventilation on arrival	471 (33)	264 (37)	207 (29)	**0.002**
Any blood transfusion on arrival	146 (10)	80 (11)	66 (9)	0.23
Type of procedure, N (%)				
Cannulation for ECMO	25 (2)	15 (2)	10 (1)	0.32
Intra-aortic balloon pump	15 (1)	4 (1)	11 (2)	0.07
IR	29 (2)	18 (3)	11 (2)	0.19
Other procedure	30 (2)	20 (3)	10 (1)	0.07
Any surgical intervention	776 (55)	364 (51)	412 (58)	**0.009**
To OR within 12 hours	324 (23)	192 (27)	142 (20)	**0.002**
Patients’ dispositions, N (%)				
Discharge home	580 (41)	269 (38)	311 (44)	
Acute rehab	327 (23)	182 (26)	145 (20)	
Skilled nursing home	253 (18)	116 (16)	137 (19)	**0.014**
Dead or hospice	218 (15)	123 (17)	95 (13)	
Other	44 (3)	22 (3)	22 (3)	

† Bold cells indicate statistically significant findings (P < 0.05).

*CCRU*, critical care resuscitation unit; *ECMO*, extracorporeal membrane oxygenation; *IQR*, interquartile range; *IR*, interventional radiology; *LOS*, length of stay; *min*, minutes; *NA*, not applicable; *OR*, operating room.

**Table 2:** Multivariate logistic regression measuring association of demographic and clinical factors with the primary outcome of transfer request to bed assignment ≤8 minutes and the secondary outcomes of transfer request to CCRU* arrival < 180 minutes and mortality.

Variables	Multivariate regression results			

	OR	95% CI LL	95% CI UL	P
Primary Outcome: Transfer Request to Bed Assignment ≤ 8minutes				
Accepting service – Stroke neurology	5.487	2.852	10.858	< 0.001
Need for OR within 12 hours	2.847	1.977	4.125	< 0.001
CCRU transfer request – Day	2.351	1.759	3.153	< 0.001
CCRU transfer request – Weekend night	2.248	1.279	3.958	0.005
Arrival SOFA score – each increment	1.036	1.001	1.073	0.047
Any OR	0.738	0.549	0.991	0.044
Accepting service – Soft tissue surgery	0.593	0.352	0.997	0.049
Secondary Outcome: Transfer Request to CCRU Arrival < 180 minutes				
Accepting service – Stroke neurology	12.814	6.092	28.510	< 0.001
Need for OR within 12 hours	3.146	2.120	4.711	< 0.001
Accepting service – Neurosurgery	2.813	1.575	5.075	< 0.001
Accepting service – Pulmonary critical care	2.415	1.098	5.411	0.029
Accepting service – Vascular surgery	1.897	1.053	3.442	0.033
Accepting service – Cardiac surgery	1.865	1.126	3.115	0.016
Any infusion on arrival	1.712	1.292	2.274	< 0.001
CCRU transfer request – Day	1.428	1.048	1.949	0.024
Serum lactate – each mmol/dL	1.080	1.011	1.156	0.025
Distance from UMMC – each kilometer	0.991	0.988	0.995	< 0.001
Arrival SOFA score – each increment	0.960	0.924	0.996	0.033
Transport type – ground	0.195	0.126	0.296	< 0.001
Any OR	0.712	0.520	0.974	0.033
Secondary Outcome: Mortality				
Accepting service – Neurosurgery	4.137	1.870	9.365	< 0.001
Accepting service – Stroke neurology	3.002	1.196	7.523	0.019
Arrival SOFA score – each increment	1.265	1.202	1.333	< 0.001
Serum lactate – each mmol/dL	1.153	1.068	1.251	< 0.001
Age – each year	1.037	1.023	1.052	< 0.001
Arrival troponin – each ng/L	1.010	1.003	1.020	0.016
Hemoglobin – each g/dL	0.875	0.804	0.950	0.002

* Only variables with significant association with the outcome of interest are reported (P < 0.05).

*CI*, confidence interval; *CCRU*, critical care resuscitation unit; *g/dL*, grams per deciliter; *LL*, lower limit 95% CI; *mmol/dL*, millimole per deciliter; *ng*, nanogram; *OR*, odds ratio; *OR*, operating room; *SOFA*, Sequential Organ Failure Assessment; *UMMC*, University of Maryland Medical Center; *UL*, upper limit 95% CI.
