# Supplementary material for: Examining Predictors of Early Admission and Transfer to the Critical Care Resuscitation Unit
Source: West J Emerg Med. 2023 Jun 28;24(4):751–62. doi: 10.5811/westjem.58356 (PMC10393446; doi:10.5811/westjem.58356)
Supplement: Supplementary file 1 [file wjem-24-751-s001.docx]

**Appendix 1**. Summary of which accepting services patients were accepted to.

| **Variables** | **All Patients** | **Immediate Bed Assignment (≤ 8 Minutes)** | **Normal Bed Assignment (> 8 Minutes)** |
| --- | --- | --- | --- |
| Total patients, N | 1422 | 712 | 710 |
| Accepting service, N (%) |  |  |  |
| Acute care emergency services | 147 (10) | 68 (10) | 79 (11) |
| Cardiac surgery | 297 (21) | 154 (22) | 143 (20) |
| Cardiology | 12 (1) | 9 (1) | 3 (1) |
| ENT | 16 (1) | 5 (1) | 11 (2) |
| General medicine | 3 (1) | 1 (1) | 2 (1) |
| Hyperbaric medicine | 1 (1) | 1 (1) | 0 (0) |
| Neurocritical care | 42 (3) | 18 (3) | 24 (3) |
| Neurosurgery | 191 (13) | 103 (14) | 88 (12) |
| Stroke neurology | 114 (8) | 87 (12) | 27 (4) |
| OB/GYN | 20 (1) | 11 (2) | 9 (1) |
| OMFS | 3 (1) | 1 (1) | 2 (1) |
| Oncology | 2 (1) | 1 (1) | 1 (1) |
| Orthopedic surgery | 24 (2) | 6 (1) | 18 (3) |
| Plastic surgery | 2 (1) | 1 (1) | 1 (1) |
| Pulmonary critical care | 57 (4) | 31 (4) | 26 (4) |
| Soft tissue surgery | 240 (16) | 91 (13) | 149 (21) |
| Surgical oncology | 13 (1) | 3 (1) | 10 (1) |
| Thoracic surgery | 25 (2) | 14 (2) | 11 (2) |
| Transplant surgery | 38 (3) | 19 (3) | 19 (3) |
| Trauma | 33 (2) | 13 (2) | 20 (3) |
| Urology | 2 (1) | 0 (0) | 2 (1) |
| Vascular surgery | 134 (9) | 69 (10) | 65 (9) |

Abbreviations: ENT, ear nose throat; OB/GYN, obstetrics and gynecology; OMFS, oral and maxillofacial surgery

**Appendix 2**. Multivariate logistic regression model measuring the association of demographic and clinical factors with the primary outcome of transfer request to bed assignment ≤ 8 minutes. All variables included in the model are reported.

| **Variables** | **Multivariate Regression Results** | | | | |
| --- | --- | --- | --- | --- | --- |
|  | **OR** | **95% CI LL** | **95% CI UL** | ***P*^†^** | **VIF** |
| **Primary Outcome: Transfer Request to Bed Assignment ≤ 8 minutes (consult to send)**^‡^ | | | | | |
| Age – each year | 0.996 | 0.988 | 1.004 | 0.36 | 1.157 |
| Sex – male | 0.844 | 0.652 | 1.091 | 0.20 | 1.102 |
| Originating hospital type – Teaching | 1.181 | 0.874 | 1.601 | 0.28 | 1.325 |
| Accepting service – Stroke neurology | 5.487 | 2.852 | 10.858 | **< 0.001** | 2.724 |
| Accepting service – Cardiology | 2.011 | 0.518 | 9.940 | 0.34 |  |
| Accepting service – Neurosurgery | 1.542 | 0.891 | 2.676 | 0.12 |  |
| Accepting service – Thoracic surgery | 1.339 | 0.516 | 3.554 | 0.55 |  |
| Accepting service – Vascular surgery | 1.204 | 0.691 | 2.101 | 0.51 |  |
| Accepting service – Cardiac surgery | 1.194 | 0.743 | 1.922 | 0.47 |  |
| Accepting service – Transplant surgery | 1.073 | 0.447 | 2.581 | 0.87 |  |
| Accepting service – Pulmonary critical care | 1.052 | 0.506 | 2.198 | 0.89 |  |
| Accepting service – OB/GYN | 0.941 | 0.301 | 3.030 | 0.92 |  |
| Accepting service – Neurocritical care | 0.808 | 0.346 | 1.853 | 0.62 |  |
| Accepting service – ENT | 0.772 | 0.165 | 3.434 | 0.73 |  |
| Accepting service – Trauma | 0.730 | 0.306 | 1.707 | 0.47 |  |
| Accepting service – Orthopedic surgery | 0.634 | 0.201 | 1.822 | 0.41 |  |
| Accepting service – Soft tissue surgery | 0.593 | 0.352 | 0.997 | **0.049** |  |
| Accepting service – Surgical oncology | 0.353 | 0.050 | 1.618 | 0.22 |  |
| CCRU transfer request – Day | 2.351 | 1.759 | 3.153 | **< 0.001** | 1.420 |
| CCRU transfer request – Weekend night | 2.248 | 1.279 | 3.958 | **0.005** | 2.824 |
| CCRU transfer request – Weekend | 0.742 | 0.487 | 1.134 | 0.17 | 2.369 |
| Distance from UMMC – each kilometer | 1.002 | 1.001 | 1.005 | 0.06 | 1.326 |
| Need for OR within 12 hours | 2.847 | 1.977 | 4.125 | **< 0.001** | 1.644 |
| Any infusion on arrival | 1.285 | 0.987 | 1.674 | 0.06 | 1.084 |
| Any IR | 0.918 | 0.385 | 2.283 | 0.85 | 1.074 |
| Any OR | 0.738 | 0.549 | 0.991 | **0.044** | 1.455 |
| Serum lactate – each mmol/dL | 1.048 | 0.984 | 1.119 | 0.15 | 1.328 |
| WBC count – each count/μL | 1.005 | 0.996 | 1.021 | 0.42 | 1.116 |
| Hemoglobin – each g/dL | 0.999 | 0.968 | 1.034 | 0.95 | 1.100 |
| Arrival troponin – each ng/L | 1.001 | 0.997 | 1.007 | 0.56 | 1.044 |
| Arrival SOFA score – each increment | 1.036 | 1.001 | 1.073 | **0.047** | 1.504 |
| Arrival shock index (HR/SBP) – each increment | 0.850 | 0.494 | 1.198 | 0.48 | 1.282 |

Abbreviations: CI, confidence interval; CCRU, Critical Care Resuscitation Unit; ENT, ear nose throat; g/dL, grams per deciliter; HR, heart rate; IR, interventional radiology; LL, lower limit 95% CI; μL, microliter; mmol/dL, millimole per deciliter; ng, nanogram; OR, odds ratio; OR, operating room; OB/GYN, obstetrics and gynecology; SOFA, Sequential Organ Failure Assessment; SBP, systolic blood pressure; UMMC, University of Maryland Medical Center; UL, upper limit 95% CI; VIF, variance inflation factor; WBC, white blood cell

**^†^**Bold cells indicate statistically significant values (*P* < 0.05)

Goodness-of-Fit Test:

^‡^Hosmer–Lemeshow Test Primary Outcome: Degrees of freedom = 8, χ^2^ = 14.55, *P* = 0.07

**Appendix 3**. Multivariate logistic regression models measuring association of demographic and clinical factors with the secondary outcomes of transfer request to CCRU arrival < 180 minutes and mortality. All variables included in the models are reported.

| **Variables** | **Multivariate Regression Results** | | | | |
| --- | --- | --- | --- | --- | --- |
|  | **OR** | **95% CI LL** | **95% CI UL** | ***P*^†^** | **VIF** |
| **Secondary Outcome: Transfer Request to CCRU Arrival < 180 minutes**^‡^ | | | | | |
| Age – each year | 0.996 | 0.987 | 1.004 | 0.33 | 1.156 |
| Sex – male | 0.994 | 0.756 | 1.308 | 0.97 | 1.095 |
| Transport type – ground | 0.195 | 0.126 | 0.296 | **< 0.001** | 1.255 |
| Originating hospital type – Teaching | 1.217 | 0.875 | 1.691 | 0.24 | 1.396 |
| Accepting service – Stroke neurology | 12.814 | 6.092 | 28.510 | **< 0.001** | 2.807 |
| Accepting service – Neurosurgery | 2.813 | 1.575 | 5.075 | **0.0005** |  |
| Accepting service – Cardiology | 2.482 | 0.598 | 11.427 | 0.22 |  |
| Accepting service – Pulmonary critical care | 2.415 | 1.098 | 5.411 | **0.029** |  |
| Accepting service – Vascular surgery | 1.897 | 1.053 | 3.442 | **0.033** |  |
| Accepting service – Cardiac surgery | 1.865 | 1.126 | 3.115 | **0.016** |  |
| Accepting service – OB/GYN | 1.829 | 0.529 | 6.599 | 0.34 |  |
| Accepting service – Neurocritical care | 1.518 | 0.636 | 3.636 | 0.35 |  |
| Accepting service – Orthopedic surgery | 1.410 | 0.463 | 4.123 | 0.53 |  |
| Accepting service – Transplant surgery | 1.340 | 0.522 | 3.379 | 0.54 |  |
| Accepting service – ENT | 1.045 | 0.237 | 4.928 | 0.95 |  |
| Accepting service – Surgical oncology | 1.017 | 0.197 | 4.314 | 0.98 |  |
| Accepting service – Soft tissue surgery | 0.685 | 0.393 | 1.191 | 0.18 |  |
| Accepting service – Trauma | 0.584 | 0.219 | 1.467 | 0.26 |  |
| Accepting service – Thoracic surgery | 0.542 | 0.177 | 1.537 | 0.26 |  |
| CCRU transfer request – Day | 1.428 | 1.048 | 1.949 | **0.024** | 1.409 |
| CCRU transfer request – Weekend night | 1.244 | 0.677 | 2.284 | 0.48 | 2.825 |
| CCRU transfer request – Weekend | 0.800 | 0.507 | 1.265 | 0.34 | 2.395 |
| Distance from UMMC – each kilometer | 0.991 | 0.988 | 0.995 | **< 0.001** | 1.539 |
| Need for OR within 12 hours | 3.146 | 2.120 | 4.711 | **< 0.001** | 1.700 |
| Any infusion on arrival | 1.712 | 1.292 | 2.274 | **0.0002** | 1.075 |
| Any IR | 1.914 | 0.650 | 7.140 | 0.28 | 1.050 |
| Any OR | 0.712 | 0.520 | 0.974 | **0.033** | 1.437 |
| Serum lactate – each mmol/dL | 1.080 | 1.011 | 1.156 | **0.025** | 1.312 |
| WBC count – each count/μL | 0.999 | 0.987 | 1.007 | 0.74 | 1.071 |
| Hemoglobin – each g/dL | 0.991 | 0.955 | 1.024 | 0.56 | 1.094 |
| Arrival shock index (HR/SBP) – each increment | 1.035 | 0.747 | 1.461 | 0.81 | 1.107 |
| Arrival troponin – each ng/L | 1.001 | 0.997 | 1.006 | 0.67 | 1.048 |
| Arrival SOFA score – each increment | 0.960 | 0.924 | 0.996 | **0.033** | 1.551 |
| **Secondary Outcome: Mortality**^‡‡^ | | | | | |
| Age – each year | 1.037 | 1.023 | 1.052 | **< 0.001** | 1.319 |
| Sex – male | 0.885 | 0.595 | 1.313 | 0.54 | 1.131 |
| Transport type – ground | 1.197 | 0.725 | 2.010 | 0.49 | 1.260 |
| Originating hospital type – Teaching | 1.264 | 0.804 | 1.984 | 0.31 | 1.318 |
| Accepting service – Neurosurgery | 4.137 | 1.870 | 9.365 | **< 0.001** | 3.755 |
| Accepting service – Cardiology | 3.281 | 0.696 | 17.184 | 0.14 |  |
| Accepting service – Stroke neurology | 3.002 | 1.196 | 7.523 | **0.019** |  |
| Accepting service – Trauma | 2.040 | 0.623 | 6.234 | 0.22 |  |
| Accepting service – Neurocritical care | 1.958 | 0.552 | 6.440 | 0.28 |  |
| Accepting service – Surgical oncology | 1.826 | 0.211 | 11.612 | 0.54 |  |
| Accepting service – Transplant surgery | 1.508 | 0.399 | 5.133 | 0.53 |  |
| Accepting service – Thoracic surgery | 1.459 | 0.355 | 5.064 | 0.57 |  |
| Accepting service – Vascular surgery | 1.197 | 0.488 | 2.857 | 0.69 |  |
| Accepting service – Cardiac surgery | 1.066 | 0.524 | 2.201 | 0.86 |  |
| Accepting service – Pulmonary critical care | 1.0001 | 0.373 | 2.602 | 0.99 |  |
| Accepting service – Orthopedic surgery | 0.499 | 0.025 | 3.147 | 0.54 |  |
| Accepting service – Soft tissue surgery | 0.492 | 0.213 | 1.120 | 0.09 |  |
| Accepting service – Other | 6E-7 | 2E-87 | 2E-145 | 0.97 |  |
| CCRU transfer request – Weekend night | 1.643 | 0.706 | 3.878 | 0.25 | 3.061 |
| CCRU transfer request – Day | 1.217 | 0.771 | 1.924 | 0.40 | 1.509 |
| CCRU transfer request – Weekend | 1.048 | 0.545 | 1.959 | 0.88 | 2.467 |
| Distance from UMMC – each kilometer | 1.003 | 0.9995 | 1.007 | 0.09 | 1.348 |
| Need for OR within 12 hours | 1.736 | 0.980 | 3.089 | 0.06 | 1.698 |
| Any infusion on arrival | 1.034 | 0.694 | 1.534 | 0.87 | 1.103 |
| Any OR | 0.915 | 0.579 | 1.437 | 0.70 | 1.496 |
| Any IR | 0.846 | 0.197 | 2.896 | 0.80 | 1.070 |
| Serum lactate – each mmol/dL | 1.153 | 1.068 | 1.251 | **< 0.001** | 1.254 |
| WBC count – each count/μL | 1.022 | 1.002 | 1.045 | 0.07 | 1.216 |
| Hemoglobin – each g/dL | 0.875 | 0.804 | 0.950 | **0.002** | 1.291 |
| Arrival SOFA score – each increment | 1.265 | 1.202 | 1.333 | **< 0.001** | 1.594 |
| Arrival shock index (HR/SBP) – each increment | 1.207 | 0.799 | 1.695 | 0.24 | 1.114 |
| Arrival troponin – each ng/L | 1.010 | 1.003 | 1.020 | **0.016** | 1.089 |
| Transfer Request to CCRU Arrival < 180 minutes | 0.754 | 0.492 | 1.153 | 0.19 | 1.310 |

Abbreviations: CI, confidence interval; CCRU, Critical Care Resuscitation Unit; ENT, ear nose throat; g/dL, grams per deciliter; HR, heart rate; IR, interventional radiology; LL, lower limit 95% CI; μL, microliter; mmol/dL, millimole per deciliter; ng, nanogram; OR, odds ratio; OR, operating room; OB/GYN, obstetrics and gynecology; SOFA, Sequential Organ Failure Assessment; SBP, systolic blood pressure; UMMC, University of Maryland Medical Center; UL, upper limit 95% CI; VIF, variance inflation factor; WBC, white blood cell

Other: indicates accepting services of OB/GYN and ENT

**^†^**Bold cells indicate statistically significant values (*P* < 0.05)

Goodness-of-Fit Tests:

^‡^Hosmer–Lemeshow Test Transfer Request to CCRU Arrival < 180 minutes: Degrees of freedom = 8, χ^2^ = 9.46, *P* = 0.30

^‡‡^Hosmer–Lemeshow Test Mortality: Degrees of freedom = 8, χ^2^ = 6.60, *P* = 0.58
